# 2-[(*E*)-2-(4-Chloro­phen­yl)ethen­yl]-1-methylpyridinium iodide monohydrate^1^

**DOI:** 10.1107/S1600536808027724

**Published:** 2008-09-06

**Authors:** Kullapa Chanawanno, Suchada Chantrapromma, Hoong-Kun Fun

**Affiliations:** a Department of Chemistry and Center of Excellence for Innovation in Chemistry, Faculty of Science, Prince of Songkla University, Hat-Yai, Songkhla 90112, Thailand; b Crystal Materials Research Unit, Department of Chemistry, Faculty of Science, Prince of Songkla University, Hat-Yai, Songkhla 90112, Thailand; c X-ray Crystallography Unit, School of Physics, Universiti Sains Malaysia, 11800 USM, Penang, Malaysia

## Abstract

In the title compound, C_14_H_13_ClN^+^·I^−^·H_2_O, the cation is nearly planar and exists in an *E* configuration; the dihedral angle between the pyridinium and benzene rings is 0.98 (17)°. The cations stack in an anti-parallel manner along the *a* axis through two π–π inter­actions between the pyridinium and benzene rings [centroid–centroid distances 3.569 (2) and 3.6818 (13) Å, respectively]. The cation, anion and water mol­ecule are linked into a chain along the *a* axis by weak C—H⋯O and C—H⋯I inter­actions together with O—H⋯I hydrogen bonds and the chains are further connected into a three-dimensional network.

## Related literature

For bond-length data, see: Allen *et al.* (1987 ▶). For related structures, see, for example: Chantrapromma *et al.* (2007*a* ▶,*b* ▶,*c* ▶). For background on non-linear optical properties, see, for example: Lakshmanaperumal *et al.* (2004 ▶); Marder *et al.* (1994 ▶); Qiu *et al.* (2007 ▶); Williams (1984 ▶); Zhai *et al.* (1999 ▶); Zhan *et al.* (2006 ▶).
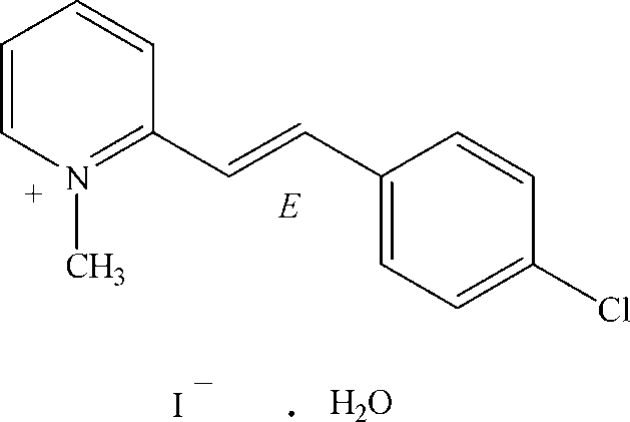


## Experimental

### 

#### Crystal data

C_14_H_13_ClN^+^·I^−^·H_2_O
*M* *_r_* = 375.62
Monoclinic, 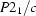

*a* = 7.0876 (1) Å
*b* = 9.8096 (2) Å
*c* = 21.0940 (4) Å
β = 95.147 (1)°
*V* = 1460.68 (5) Å^3^
*Z* = 4
Mo *K*α radiation
μ = 2.36 mm^−1^
*T* = 100.0 (1) K
0.28 × 0.17 × 0.07 mm

#### Data collection

Bruker SMART APEXII CCD area-detector diffractometer
Absorption correction: multi-scan (**SADABS**; Bruker, 2005 ▶) *T*_min_ = 0.560, *T*_max_ = 0.845
18928 measured reflections
4241 independent reflections
3486 reflections with *I* > 2σ(*I*)
*R*_int_ = 0.038

#### Refinement

*R*[*F* ^2^ > 2σ(*F* ^2^)] = 0.037
*wR*(*F* ^2^) = 0.104
*S* = 1.13
4241 reflections
164 parameters
H-atom parameters constrained
Δρ_max_ = 2.13 e Å^−3^
Δρ_min_ = −0.79 e Å^−3^

### 

Data collection: *APEX2* (Bruker, 2005 ▶); cell refinement: *APEX2*; data reduction: *SAINT* (Bruker, 2005 ▶); program(s) used to solve structure: *SHELXTL* (Sheldrick, 2008 ▶); program(s) used to refine structure: *SHELXTL*; molecular graphics: *SHELXTL*; software used to prepare material for publication: *SHELXTL* and *PLATON* (Spek, 2003 ▶).

## Supplementary Material

Crystal structure: contains datablocks global, I. DOI: 10.1107/S1600536808027724/is2329sup1.cif

Structure factors: contains datablocks I. DOI: 10.1107/S1600536808027724/is2329Isup2.hkl

Additional supplementary materials:  crystallographic information; 3D view; checkCIF report

## Figures and Tables

**Table 1:** Hydrogen-bond geometry (Å, °)

*D*—H⋯*A*	*D*—H	H⋯*A*	*D*⋯*A*	*D*—H⋯*A*
O1*W*—H1*W*1⋯I1	0.86	2.87	3.592 (4)	143
O1*W*—H2*W*1⋯I1^i^	0.85	2.87	3.567 (4)	141
C14—H14*A*⋯O1*W*	0.96	2.50	3.202 (5)	130
C14—H14*D*⋯O1*W*^ii^	0.96	2.56	3.460 (5)	157
C1—H1*A*⋯I1^iii^	0.93	3.20	3.830 (4)	127
C2—H2*A*⋯I1^iv^	0.93	3.17	3.825 (4)	129
C3—H3*A*⋯I1^iv^	0.93	3.21	3.840 (4)	127

Symmetry codes: (i) 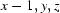; (ii) 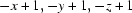; (iii) 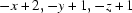; (iv) 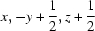.

**Fig. 1.**
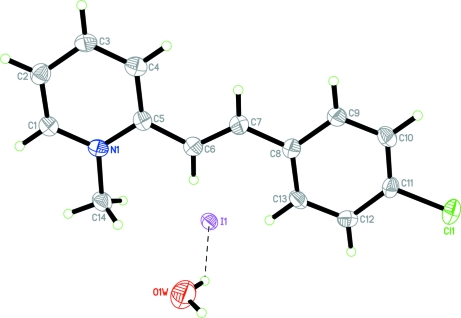
The title compound showing 50% probability displacement ellipsoids and the atom-numbering scheme. The O—H···I hydrogen bond was drawn as dashed line.

**Fig. 2.**
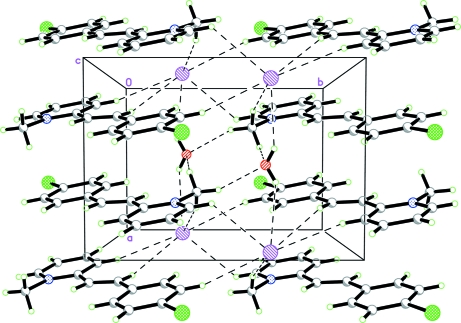
The packing diagram of the title structure viewed approximately along the c axis. Hydrogen bonds were drawn as dashed lines.

## supplementary crystallographic information

### Comment

In the last two decades, many efforts were focused on the discovery of new organic materials which exhibit large nonlinear optical (NLO) properties and would have applications in the fields of optoelectronics and photonics (Lakshmanaperumal *et al.*, 2004; Marder *et al.*, 1994; Qiu *et al.*, 2007; Zhai *et al.*, 1999; Zhan *et al.*, 2006). In order to obtain second-order NLO single crystals, the main requirements should be the choice of molecules with large hyperpolarizability (*β*) and the alignment of these molecules with optimal orientation into a noncentrosymmetric space group in the crystal (Williams, 1984). Among the known organic NLO materials, ionic chromophores are of great interest because they exhibit large first hyperpolarizabilities (*β*) and have high melting points and hardness of their crystals. At the molecular level, a generally popular approach towards NLO materials is to design and synthesize compounds with extended conjugated π-systems with donor and acceptor groups because such compounds are likely to exhibit large values of molecular hyperpolarizability (*β*) and to possess polarization. Styryl pyridinium derivatives are considered to be good conjugated π-systems. In continuation of our on-going research on nonlinear optical materials (Chantrapromma *et al.*, 2007*a*,*b*,*c*), the title compound, (I), was synthesized and the X-ray structure analysis was carried out in order to obtain detailed information about the molecular packing. However, compound (I) crystallizes in monoclinic space group *P*2_1_/*c* and doesn't exhibit second-order nonlinear optic properties.

The asymmetric unit of the title compound consists of C_14_H_13_ClN^+^ cation, I^-^ anion and one water molecule (Fig. 1). The conformation of the cation is essentially planar as indicated by the dihedral angle between the pyridinium (N1/C1—C5) and the benzene (C8—C13) rings, being 0.98 (17)°. The mean plane through C5/C6/C7/C8 plane makes dihedral angles of 6.1 (4)° and 6.4 (4)° with pyridinium and benzene rings, respectively. The cation exists in the *E* configuration and the torsion angle C5—C6—C7—C8 = -179.2 (3)°. The bond distances and angles in (I) have normal values (Allen *et al.*, 1987) and comparable with closely related structures (Chantrapromma *et al.*, 2007*a*,*b*,*c*).

The packing of the molecule down the *c* axis (Fig. 2), showing that the cation is linked with water molecule by weak C—H···O interactions (Table 1) and linked with I^-^ anions by weak C—H···I interactions (Table 1) whereas the I^-^ anion is linked with water molecule by O—H···I hydrogen bonds, forming one-dimensional chains along the *a* axis. These chains are further connected into a three-dimensional network (Fig. 2). π···π interactions involving pyridinium and benzene rings were also observed with *Cg*_1_···*Cg*_2_ distances of 3.662 (2) Å (symmetry code; 1 - *x*, -*y*, 1 - *z*) and 3.569 (2) Å (symmetry code; 2 - *x*, -*y*, 1 - *z*); *Cg*_1_ and *Cg*_2_ are the centroids of the N1/C1–C5 pyridinium and C8–C13 benzene rings, respectively. The crystal is stabilized by O—H···I hydrogen bond, weak C—H···O and C—H···I interactions.

### Experimental

The title compound was prepared by mixing solutions of 1,2-dimethylpyridinium iodide, 4-chlorobenzaldehyde and piperidine (1:1:1 molar ratio) in methanol. The resulting solution was refluxed for 12 hr under a nitrogen atmosphere. The solid which formed was filtered and washed with chloroform. Orange plate-like single-crystal suitable for X-ray diffraction analysis was obtained by recrystallization from methanol by slow evaporation of the solvent at ambient temperature after several days, Mp. 492–493 K.

### Refinement

All H atoms were placed in calculated positions (O—H = 0.85–0.86 and C—H = 0.93–0.96 Å) and refined as riding, with *U*_iso_(H) = 1.2*U*_eq_(C) or 1.5*U*_eq_(O, methyl C), A rotating group model was used for the methyl groups. The highest residual electron density peak is located at 0.76 Å from I1 and the deepest hole is located at 0.59 Å from I1.

### Crystal data

**Table:**

C_14_H_13_ClN^+^·I^−^·H_2_O	*F*(000) = 736
*M**_r_* = 375.62	*D*_x_ = 1.708 Mg m^−^^3^
Monoclinic, *P*2_1_/*c*	Melting point: 492-493 K K
Hall symbol: -P 2ybc	Mo *K*α radiation, λ = 0.71073 Å
*a* = 7.0876 (1) Å	Cell parameters from 4241 reflections
*b* = 9.8096 (2) Å	θ = 1.9–30.0°
*c* = 21.0940 (4) Å	µ = 2.36 mm^−^^1^
β = 95.147 (1)°	*T* = 100 K
*V* = 1460.68 (5) Å^3^	Plate, orange
*Z* = 4	0.28 × 0.17 × 0.07 mm

### Data collection

**Table:**

Bruker SMART APEXII CCD area-detector diffractometer	4241 independent reflections
Radiation source: fine-focus sealed tube	3486 reflections with *I* > 2σ(*I*)
graphite	*R*_int_ = 0.038
Detector resolution: 8.33 pixels mm^-1^	θ_max_ = 30.0°, θ_min_ = 1.9°
ω scans	*h* = −9→9
Absorption correction: multi-scan (SADABS; Bruker, 2005)	*k* = −13→12
*T*_min_ = 0.561, *T*_max_ = 0.845	*l* = −26→29
18928 measured reflections	

### Refinement

**Table:**

Refinement on *F*^2^	Primary atom site location: structure-invariant direct methods
Least-squares matrix: full	Secondary atom site location: difference Fourier map
*R*[*F*^2^ > 2σ(*F*^2^)] = 0.037	Hydrogen site location: inferred from neighbouring sites
*wR*(*F*^2^) = 0.104	H-atom parameters constrained
*S* = 1.13	*w* = 1/[σ^2^(*F*_o_^2^) + (0.0483*P*)^2^ + 1.1431*P*] where *P* = (*F*_o_^2^ + 2*F*_c_^2^)/3
4241 reflections	(Δ/σ)_max_ = 0.001
164 parameters	Δρ_max_ = 2.13 e Å^−^^3^
0 restraints	Δρ_min_ = −0.79 e Å^−^^3^

### Special details

**Table:**

Experimental. The data was collected with the Oxford Cyrosystem Cobra low-temperature attachment.
Geometry. All e.s.d.'s (except the e.s.d. in the dihedral angle between two l.s. planes) are estimated using the full covariance matrix. The cell e.s.d.'s are taken into account individually in the estimation of e.s.d.'s in distances, angles and torsion angles; correlations between e.s.d.'s in cell parameters are only used when they are defined by crystal symmetry. An approximate (isotropic) treatment of cell e.s.d.'s is used for estimating e.s.d.'s involving l.s. planes.
Refinement. Refinement of *F*^2^ against ALL reflections. The weighted *R*-factor *wR* and goodness of fit *S* are based on *F*^2^, conventional *R*-factors *R* are based on *F*, with *F* set to zero for negative *F*^2^. The threshold expression of *F*^2^ > σ(*F*^2^) is used only for calculating *R*-factors(gt) *etc*. and is not relevant to the choice of reflections for refinement. *R*-factors based on *F*^2^ are statistically about twice as large as those based on *F*, and *R*- factors based on ALL data will be even larger.

### Fractional atomic coordinates and isotropic or equivalent isotropic displacement parameters (Å^2^)

**Table:**

	*x*	*y*	*z*	*U*_iso_*/*U*_eq_	
I1	0.96440 (3)	0.30947 (2)	0.374487 (10)	0.02517 (9)	
Cl1	0.65015 (14)	−0.33018 (11)	0.25791 (5)	0.0349 (2)	
N1	0.7517 (4)	0.2927 (3)	0.58820 (14)	0.0228 (6)	
O1W	0.4685 (5)	0.3289 (4)	0.39663 (18)	0.0589 (10)	
H1W1	0.5617	0.2949	0.3782	0.088*	
H2W1	0.3691	0.2881	0.3808	0.088*	
C1	0.7754 (5)	0.3535 (4)	0.64637 (17)	0.0254 (7)	
H1A	0.7521	0.4464	0.6498	0.030*	
C2	0.8329 (5)	0.2805 (4)	0.69966 (18)	0.0277 (8)	
H2A	0.8473	0.3228	0.7393	0.033*	
C3	0.8698 (5)	0.1412 (4)	0.69383 (17)	0.0262 (7)	
H3A	0.9115	0.0900	0.7294	0.031*	
C4	0.8440 (5)	0.0817 (4)	0.63559 (17)	0.0264 (7)	
H4A	0.8674	−0.0111	0.6318	0.032*	
C5	0.7828 (5)	0.1569 (4)	0.58078 (17)	0.0221 (7)	
C6	0.7492 (5)	0.0969 (4)	0.51828 (17)	0.0256 (7)	
H6A	0.7187	0.1548	0.4839	0.031*	
C7	0.7592 (5)	−0.0353 (4)	0.50694 (17)	0.0268 (7)	
H7A	0.7883	−0.0912	0.5421	0.032*	
C8	0.7293 (5)	−0.1035 (4)	0.44465 (16)	0.0235 (7)	
C9	0.7663 (5)	−0.2435 (4)	0.44164 (18)	0.0266 (7)	
H9A	0.8075	−0.2903	0.4786	0.032*	
C10	0.7425 (5)	−0.3135 (4)	0.38414 (19)	0.0271 (8)	
H10A	0.7680	−0.4063	0.3824	0.033*	
C11	0.6808 (5)	−0.2432 (4)	0.33021 (17)	0.0238 (7)	
C12	0.6421 (5)	−0.1050 (4)	0.33059 (17)	0.0258 (7)	
H12A	0.6005	−0.0596	0.2932	0.031*	
C13	0.6668 (5)	−0.0354 (4)	0.38847 (17)	0.0250 (7)	
H13A	0.6414	0.0575	0.3896	0.030*	
C14	0.6940 (5)	0.3805 (4)	0.53292 (18)	0.0298 (8)	
H14D	0.6802	0.4727	0.5470	0.045*	
H14A	0.5754	0.3489	0.5126	0.045*	
H14B	0.7889	0.3769	0.5032	0.045*	

### Atomic displacement parameters (Å^2^)

**Table:**

	*U*^11^	*U*^22^	*U*^33^	*U*^12^	*U*^13^	*U*^23^
I1	0.02744 (14)	0.02228 (14)	0.02596 (14)	0.00264 (9)	0.00324 (9)	0.00111 (9)
Cl1	0.0368 (5)	0.0385 (6)	0.0293 (5)	−0.0051 (4)	0.0027 (4)	−0.0126 (4)
N1	0.0206 (13)	0.0229 (16)	0.0249 (15)	0.0010 (11)	0.0027 (11)	0.0046 (12)
O1W	0.0347 (17)	0.083 (3)	0.058 (2)	0.0104 (16)	−0.0028 (15)	−0.028 (2)
C1	0.0246 (17)	0.0238 (18)	0.0278 (18)	0.0003 (14)	0.0028 (14)	0.0000 (15)
C2	0.0255 (17)	0.032 (2)	0.0261 (18)	−0.0004 (14)	0.0028 (14)	−0.0020 (15)
C3	0.0209 (16)	0.031 (2)	0.0263 (17)	0.0017 (14)	−0.0021 (13)	0.0030 (15)
C4	0.0218 (16)	0.0267 (19)	0.0307 (18)	−0.0010 (14)	0.0017 (14)	0.0034 (15)
C5	0.0175 (15)	0.0230 (18)	0.0265 (17)	−0.0009 (12)	0.0050 (12)	−0.0007 (14)
C6	0.0265 (17)	0.0245 (19)	0.0258 (17)	0.0002 (14)	0.0018 (14)	0.0001 (14)
C7	0.0272 (17)	0.0261 (19)	0.0268 (18)	0.0021 (14)	0.0002 (14)	−0.0019 (15)
C8	0.0227 (16)	0.0244 (19)	0.0236 (16)	−0.0012 (13)	0.0027 (13)	−0.0021 (14)
C9	0.0288 (18)	0.0231 (19)	0.0272 (18)	0.0000 (14)	−0.0009 (14)	0.0014 (15)
C10	0.0266 (17)	0.0195 (18)	0.035 (2)	−0.0016 (14)	0.0038 (15)	0.0009 (15)
C11	0.0203 (16)	0.0253 (19)	0.0257 (17)	−0.0029 (13)	0.0025 (13)	−0.0064 (14)
C12	0.0233 (16)	0.027 (2)	0.0267 (17)	0.0026 (14)	−0.0011 (13)	0.0029 (14)
C13	0.0246 (16)	0.0172 (17)	0.0329 (19)	0.0022 (13)	0.0013 (14)	0.0009 (14)
C14	0.037 (2)	0.025 (2)	0.0281 (18)	0.0052 (15)	0.0056 (15)	0.0032 (15)

### Geometric parameters (Å, °)

**Table:**

Cl1—C11	1.744 (4)	C6—H6A	0.9300
N1—C1	1.361 (5)	C7—C8	1.473 (5)
N1—C5	1.361 (5)	C7—H7A	0.9300
N1—C14	1.478 (5)	C8—C13	1.397 (5)
O1W—H1W1	0.8628	C8—C9	1.400 (5)
O1W—H2W1	0.8523	C9—C10	1.391 (5)
C1—C2	1.364 (5)	C9—H9A	0.9300
C1—H1A	0.9300	C10—C11	1.368 (5)
C2—C3	1.399 (6)	C10—H10A	0.9300
C2—H2A	0.9300	C11—C12	1.383 (5)
C3—C4	1.358 (5)	C12—C13	1.396 (5)
C3—H3A	0.9300	C12—H12A	0.9300
C4—C5	1.407 (5)	C13—H13A	0.9300
C4—H4A	0.9300	C14—H14D	0.9600
C5—C6	1.444 (5)	C14—H14A	0.9600
C6—C7	1.322 (5)	C14—H14B	0.9600
			
C1—N1—C5	121.6 (3)	C13—C8—C9	118.5 (3)
C1—N1—C14	117.3 (3)	C13—C8—C7	123.3 (3)
C5—N1—C14	121.0 (3)	C9—C8—C7	118.2 (3)
H1W1—O1W—H2W1	106.3	C10—C9—C8	121.0 (3)
N1—C1—C2	121.1 (4)	C10—C9—H9A	119.5
N1—C1—H1A	119.4	C8—C9—H9A	119.5
C2—C1—H1A	119.4	C11—C10—C9	118.8 (3)
C1—C2—C3	119.0 (4)	C11—C10—H10A	120.6
C1—C2—H2A	120.5	C9—C10—H10A	120.6
C3—C2—H2A	120.5	C10—C11—C12	122.5 (3)
C4—C3—C2	119.2 (3)	C10—C11—Cl1	119.1 (3)
C4—C3—H3A	120.4	C12—C11—Cl1	118.4 (3)
C2—C3—H3A	120.4	C11—C12—C13	118.4 (3)
C3—C4—C5	121.7 (4)	C11—C12—H12A	120.8
C3—C4—H4A	119.2	C13—C12—H12A	120.8
C5—C4—H4A	119.2	C12—C13—C8	120.9 (3)
N1—C5—C4	117.4 (3)	C12—C13—H13A	119.6
N1—C5—C6	119.3 (3)	C8—C13—H13A	119.6
C4—C5—C6	123.4 (3)	N1—C14—H14D	109.5
C7—C6—C5	123.9 (3)	N1—C14—H14A	109.5
C7—C6—H6A	118.0	H14D—C14—H14A	109.5
C5—C6—H6A	118.0	N1—C14—H14B	109.5
C6—C7—C8	127.0 (4)	H14D—C14—H14B	109.5
C6—C7—H7A	116.5	H14A—C14—H14B	109.5
C8—C7—H7A	116.5		
			
C5—N1—C1—C2	0.5 (5)	C5—C6—C7—C8	−179.2 (3)
C14—N1—C1—C2	−178.4 (3)	C6—C7—C8—C13	−6.4 (6)
N1—C1—C2—C3	0.7 (5)	C6—C7—C8—C9	173.2 (4)
C1—C2—C3—C4	−1.3 (5)	C13—C8—C9—C10	0.3 (5)
C2—C3—C4—C5	0.6 (5)	C7—C8—C9—C10	−179.4 (3)
C1—N1—C5—C4	−1.1 (5)	C8—C9—C10—C11	−0.4 (5)
C14—N1—C5—C4	177.7 (3)	C9—C10—C11—C12	0.3 (5)
C1—N1—C5—C6	177.9 (3)	C9—C10—C11—Cl1	−179.9 (3)
C14—N1—C5—C6	−3.2 (5)	C10—C11—C12—C13	−0.2 (5)
C3—C4—C5—N1	0.6 (5)	Cl1—C11—C12—C13	−179.9 (3)
C3—C4—C5—C6	−178.4 (3)	C11—C12—C13—C8	0.0 (5)
N1—C5—C6—C7	−173.9 (3)	C9—C8—C13—C12	−0.1 (5)
C4—C5—C6—C7	5.1 (6)	C7—C8—C13—C12	179.6 (3)

### Hydrogen-bond geometry (Å, °)

**Table:**

*D*—H···*A*	*D*—H	H···*A*	*D*···*A*	*D*—H···*A*
O1W—H1W1···I1	0.86	2.87	3.592 (4)	143
O1W—H2W1···I1^i^	0.85	2.87	3.567 (4)	141
C14—H14A···O1W	0.96	2.50	3.202 (5)	130
C14—H14D···O1W^ii^	0.96	2.56	3.460 (5)	157
C1—H1A···I1^iii^	0.93	3.20	3.830 (4)	127
C2—H2A···I1^iv^	0.93	3.18	3.825 (4)	129
C3—H3A···I1^iv^	0.93	3.21	3.840 (4)	127

Symmetry codes:  (i) *x*−1, *y*, *z*; (ii) −*x*+1, −*y*+1, −*z*+1; (iii) −*x*+2, −*y*+1, −*z*+1; (iv) *x*, −*y*+1/2, *z*+1/2.
